# Evaluation of activated irrigation techniques for the removal of calcium hydroxide from simulated internal root resorption cavities: an in vitro comparative study

**DOI:** 10.1186/s12903-026-09569-9

**Published:** 2026-08-27

**Authors:** Amira Shafie Ibrahim Mohamed, Dalia Mohamed Mukhtar Fayyad, Dalia Abd-Allah Mohamed

**Affiliations:** 1 Department of Endodontics, Faculty of Dentistry, Suez Canal University, Ismailia, Egypt; 2 Department of Endodontics, Faculty of Dentistry, Sinai University, Kantara-Branch, Ismailia, Egypt

**Keywords:** Calcium hydroxide, Internal root resorption, Irrigation activation, Sonic irrigation, Ultrasonic irrigation, XP‑endo Finisher

## Abstract

**Background:**

This study aimed to compare the calcium hydroxide [Ca(OH)₂] removal efficiency of six irrigation‑activation techniques and conventional syringe irrigation (control) from simulated internal root‑resorptive cavities in vitro.

**Materials and methods:**

Seventy single-rooted human teeth were standardized to a length of 18 mm, prepared, and split longitudinally. A standardized hemispherical resorptive cavity was created in each root half; the halves were then reassembled and filled with premixed Ca(OH)₂. Specimens were incubated for 7 days and randomly allocated (*n* = 10) to seven groups: (1) conventional syringe irrigation (control), (2) manual dynamic agitation (MDA), (3) XP‑Endo Finisher, (4) sonic EDDY, (5) sonic EQ‑S, (6) ultrasonic Irrisafe, or (7) ultrasonic Ultra X. In each irrigation protocol, two 30 s activations with 10 mL 2.5% NaOCl were used followed by one 30 s activation with 5 mL 17% EDTA. Residual Ca(OH)₂ percentages (%) within the simulated resorptive cavity (primary outcome) and the entire root canal system, including the coronal, middle, and apical thirds (secondary outcomes) was quantified stereo-microscopically using ImageJ software. Data were analyzed using Kruskal-Wallis and Friedman tests with Dunn–Bonferroni post hoc comparisons (α = 0.05).

**Results:**

Significant differences were observed among the tested groups (*P* ≤ 0.001). Within the simulated resorptive cavities, EDDY group demonstrated the significantly lowest percentage values of residual Ca(OH)₂ [median: 1.48%, IQR (0.65–1.59)] (*P* < 0.05) followed by XP-endo Finisher [median: 23.31%, IQR (16.61–24.83)] and Irrisafe [median: 36.34%, IQR (14.31–49.05)] groups. EDDY, XP-Endo Finisher and Irrisafe groups showed the significantly lowest overall (total) percentage values (*P* < 0.05), with no significant differences among them. Most irrigation protocols tested showed significant lower percentage values in the coronal third compared with the apical third, while no significant difference was detected between root thirds within the EDDY and Ultra X groups (*P* = 0.199, *P* = 0.182). None of the tested techniques achieved complete removal of Ca(OH)₂.

**Conclusions:**

Activated irrigation significantly enhances Ca(OH)₂ retrieval from internal resorptive defects; however, complete removal is not achieved with any of the tested techniques. EDDY demonstrates superior efficacy in removing Ca(OH)₂ specifically from simulated internal root resorption cavities. Across the entire root canal system, EDDY, XP-Endo Finisher, and Irrisafe demonstrate the highest overall efficacy for Ca(OH)₂ removal.

## Background

Internal root resorption (IRR) represents a unique endodontic pathology characterized by progressive, clastic-cell-mediated dentinal loss initiated by pulpal inflammation secondary to trauma or infection [1]. Early resorptive lesions are typically asymptomatic dentinal cavities and they are detected radiographically as round or oval radiolucencies. Left unchecked, IRR may perforate the root, complicate disinfection and compromise tooth survival. Treatment involves meticulous removal of infected granulation tissue and suppression of resorptive cellular activity to arrest further dentinal degradation [2].

Calcium hydroxide [Ca(OH)₂] remains the medicament of choice for such cases due to its high alkalinity (pH ~ 12.5), potent antimicrobial properties, and capacity to neutralize and inhibit osteoclastic activities, thereby promoting the repair process within resorptive lesions [1, 3, 4]. However, instrumentation alone often fails to adequately clean these complex cavities, resulting in residual Ca (OH)₂ that could adversely affect the physical and mechanical properties of dentin. Research has shown that the mechanical integrity of the tooth structure is weakened by incomplete intracanal medication removal, making the endodontically treated tooth more prone to fracture and eventual failure [5]. Moreover, these remnants may compromise sealer penetration and impair dentin-sealer bonding. This eventually reduces the root canal filling materials’ capacity to seal [6]. These potential adverse effects underscore the need for effective irrigation strategies capable of minimizing residual medicament within the root canal system prior to obturation.

Side-vented needles used in traditional syringe irrigation deliver positive-pressure flow, but their effectiveness is hampered by vapor-lock formation, apical air retention and limited irrigant exchange, particularly within resorptive niches [7]. Consequently, various irrigation activation techniques have emerged aiming to enhance fluid movement through distinct energy transfer mechanisms with the purpose of optimizing irrigant distribution, facilitating intracanal medication removal, and overcoming the hydrodynamic limitations of passive irrigation protocols [8–14].

Manual dynamic agitation (MDA) relies on repetitive apico-coronal pumping (approximately two mm amplitude) of a master gutta-percha cone to generate transient pressure gradients, thereby improving bulk irrigant exchange via piston-like hydrodynamic shear effects [14]. The XP-Endo Finisher file, by contrast, exploits the shape-memory and super-elastic properties of nickel–titanium (Ni-Ti) alloy to achieve adaptive three-dimensional expansion within canal irregularities during rotation (800–1,000 rpm). This mechanism primarily includes mechanical disruption of stagnant irrigation, accompanied by secondary microstreaming effects, potentially enhancing medication detachment [10]. Sonic activation systems such as EDDY and EQ-S operate at relatively low frequencies (e.g., approximately 6 kHz for EDDY) using flexible polymer tips, producing vigorous high -amplitude oscillations that generate acoustic streaming and cavitation-like phenomena in irrigant media, thereby enhancing shear forces and potentially improving medication removal within resorptive defects [8, 11]. On the other hand, passive ultrasonic irrigation (PUI) systems, using devices such as Irrisafe and Ultra X, operate at higher frequencies (approximately 25–45 kHz), producing more intense acoustic microstreaming and cavitation effects due to rapid oscillation of the metallic tip within a confined fluid environment, which theoretically enhances irrigant penetration and debridement efficiency in anatomically complex regions. However, the rigidity of the metallic tip may limit access to inaccessible canal regions, and this issue remains a matter of debate in the literature [12, 13]. Taken together, these systems differ fundamentally in their energy source (manual, mechanical, sonic, ultrasonic), oscillation frequency, amplitude, and ability to adapt to irregular canal anatomy. These mechanistic differences provide a theoretical basis for expecting variability in the effectiveness of calcium hydroxide removal from resorptive cavities and other regions of the root canal system.

Although previous studies have investigated the efficacy of various irrigation-activation techniques for calcium hydroxide removal from artificial grooves and simulated internal resorption defects, the findings have been inconsistent, and several important limitations remain. First, many previous studies relied on qualitative or semi-quantitative scoring systems in their investigations, which may be subject to observer interpretation and provide limited discrimination between techniques. In contrast, quantitative image-based analysis allows a more objective and sensitive assessment of residual medicament. Second, most studies focused primarily on calcium hydroxide removal from the simulated resorptive cavity itself, without considering residual material in the remaining root canal regions. Because effective debridement of the entire root canal system is essential, evaluating both the resorptive defect and the surrounding canal regions may provide a more comprehensive and clinically relevant assessment of irrigation efficacy. Furthermore, no single study has comprehensively compared conventional syringe irrigation, manual dynamic agitation, XP-endo Finisher, sonic activation (EDDY and EQ-S), and ultrasonic activation (Irrisafe and Ultra X) under standardized experimental conditions. Accordingly, the present in vitro study was designed to address this knowledge gap by quantitatively evaluating and comparing the efficacy of these seven contemporary irrigation approaches in removing calcium hydroxide from simulated internal root resorption defects as the primary outcome, and from the overall root canal regions (coronal, middle, and apical) as secondary outcomes.

The primary null hypothesis was that no statistically significant differences would exist among the seven tested irrigation techniques in calcium hydroxide removal from the simulated internal resorptive cavity. A secondary null hypothesis was that no statistically significant differences would exist among the tested techniques regarding calcium hydroxide removal from the overall root canal system.

## Materials and methods (Fig. 1)

**Fig. 1:**
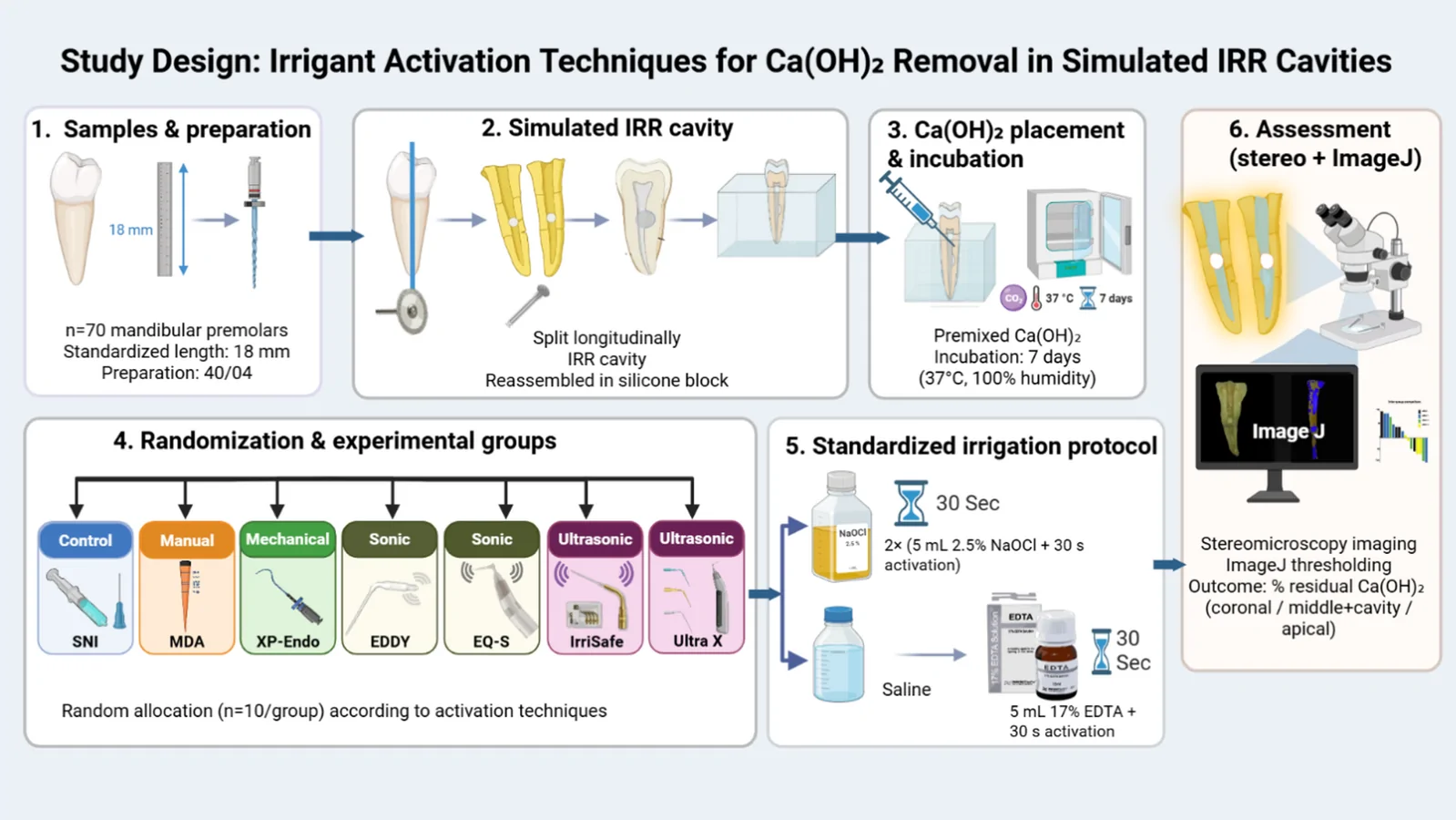
Graphical abstract for the current study methodology

### Determination of sample size

The required sample size was determined a priori using G*Power software (Version 3.1.9.7; Heinrich Heine University Düsseldorf, Germany) [15]. The sample size was estimated using the ANOVA framework (F-test family) to power the primary omnibus comparison among the seven independent experimental groups, which is commonly used approach for prospective sample size estimation irrespective of the final statistical test selected after assessing data distribution. The planning effect size (Cohen’s *f* = 0.47) was derived from the published group means and standard deviations for the percentage of remaining calcium hydroxide reported in comparable in vitro studies evaluating different irrigation activation techniques [16, 17]. Assuming a two-sided significance level of α = 0.05 and a statistical power of 80% (1 − β = 0.80), the required sample size was calculated to be 70 specimens (10 per group). This sample size allocation is consistent with previous in vitro studies evaluating calcium hydroxide removal using different irrigation activation protocols [16, 18, 19].

### Sample selection and criteria

This double-blind in vitro study was conducted on seventy unidentified, freshly extracted, single-rooted human mandibular premolars following approval from the Research Ethics Committee of the Faculty of Dentistry, Suez Canal University (REC No. 434/2022), Egypt. All experiments were performed in accordance with relevant guidelines and regulations, including the Declaration of Helsinki.The teeth used in this study were collected from the Department of Oral and Maxillofacial Surgery, Faculty of Dentistry, Suez Canal University. The extracted teeth were immediately disinfected by sodium hypochlorite 5.25% immersion for 10 min, subsequently washed and stored in distilled water (DEXA Co., Cairo, Egypt) until use. All teeth were used within one week of extraction, and the distilled water was renewed daily during the storage period.

Only single-rooted teeth with sound structure, mature apical morphology, and length ranging from 19 to 21 mm were included. Specimens were screened using digital periapical radiographs (FONA Dental, Assago, Milan, Italy) obtained in both buccolingual and mesiodistal projections to confirm the presence of a single straight canal (Vertucci Type I) [20] and the absence of internal resorption, calcification or previous endodontic treatment [21]. To further standardize canal anatomy, canal dimensions were assessed at the middle third, and excessively oval canals, defined as those with a buccolingual-to-mesiodistal diameter ratio greater than 2, were excluded. Teeth with canal curvatures exceeding 10° as determined by the Schneider’s method [22] were also excluded. A dental operating microscope (DOM; OMS3200, Zumax Medical Co., Ltd., Suzhou, China; ×25 magnification) was used to exclude teeth exhibiting cracks, root caries or external resorption.

### Specimen preparation

Following debridement and disinfection, crowns were reduced with a diamond disc (Kangda Dental Abrasives, Anyang, China) to achieve a standardize teeth length of 18 mm and to ensure straight-line access [23]. High-speed fissure burs under water cooling were used to create endodontic access cavities. Working length (WL) was calculated by using a stereomicroscope to view a size 15 K file (Mani Inc., Utsunomiya, Japan) at the apical foramen and deducting 1 mm.

The initial apical binding file size was verified to be #20 in all teeth (reach passively to WL without passing beyond the apical foramen) to standardize preoperative apical canal dimensions; any specimen exhibiting a smaller or larger initial file size was excluded and replaced with another. Chemo-mechanical preparation was performed using VDEYA rotary Ni-Ti- instruments (Huizhou Videya Technology Co., Ltd., Guangdong, China) in a crown-down sequence (orifice opener, 25/04, 30/04, 35/04 and 40/04) driven by a Woodpecker Endo motor at 300 rpm and 2 N·cm torque. A glide path was established with #10 K-files (MANI, INC. Industrial Park, Japan) using five mL 2.5% sodium hypochlorite (NaOCl, DEXA Co., Cairo, Egypt) between files. Each file change was followed by irrigation with five mL 2.5% NaOCl delivered through a 30-gauge side-vented needle (Fanta Dental, Shanghai, China) positioned two mm short of the WL. Canals were rinsed with five mL 17% ethylenediaminetetraacetic acid (EDTA, JK Dental Vision, Egypt) to remove smear layer after shaping. A 5-mL volume of saline solution (0.9% NaCl; Egyptian Company for Dental and Medical Supplies, Cairo, Egypt) was used following irrigation with NaOCl and EDTA to remove any residual irrigants and prevent potential carry-over effects. Subsequently, the root canals were dried using size 40/0.04 absorbent paper points (Meta biomed, Korea).

### Mounting and simulation of internal resorption

Glass ionomer temporary filling material (Medicem forte lx, Germany) and Teflon tape were used to temporarily close each tooth’s coronal access. Teeth were mounted vertically in individual silicone impression blocks (2 × 2 × 2 cm) using silicone impression material (Coltene, Langenau, Germany) to create a closed irrigation system. After silicone polymerization, the specimens were taken out and longitudinally sectioned using a diamond disk to create grooves on the mesial and distal surfaces (IsoMet 4000, Buehler, Illinois, USA), then splitting with a wax carver blade. Each root half was inspected by stereomicroscope equipped with a digital camera (MZ12.5, Leica microsystems, Germany) and digital caliper (Corning, USA). Root halves having cracks, extreme internal anatomical variations in canal dimensions, ovality, dentin thickness or could not be reassembled without gaps were replaced.

The standardized site of the resorption cavity in each half was determined by a digital caliper at the canal’s middle third (at a distance of six mm from the tooth apex). Standardized hemispherical artificial resorptive cavities (1.8 mm diameter, 0.9 mm depth) were prepared using a #31/0.018 ISO diamond round bur (MANI, INC., Industrial Park, Utsunomiya, Tochigi, Japan) in a high-speed operated handpiece under water coolant. Dimensional consistency was verified using a digital caliper and photographs taken using stereomicroscope at ×10 magnification to ensure uniformity across all specimens (≈ 2.55 mm^2^ surface area).

**Fig. 2:**
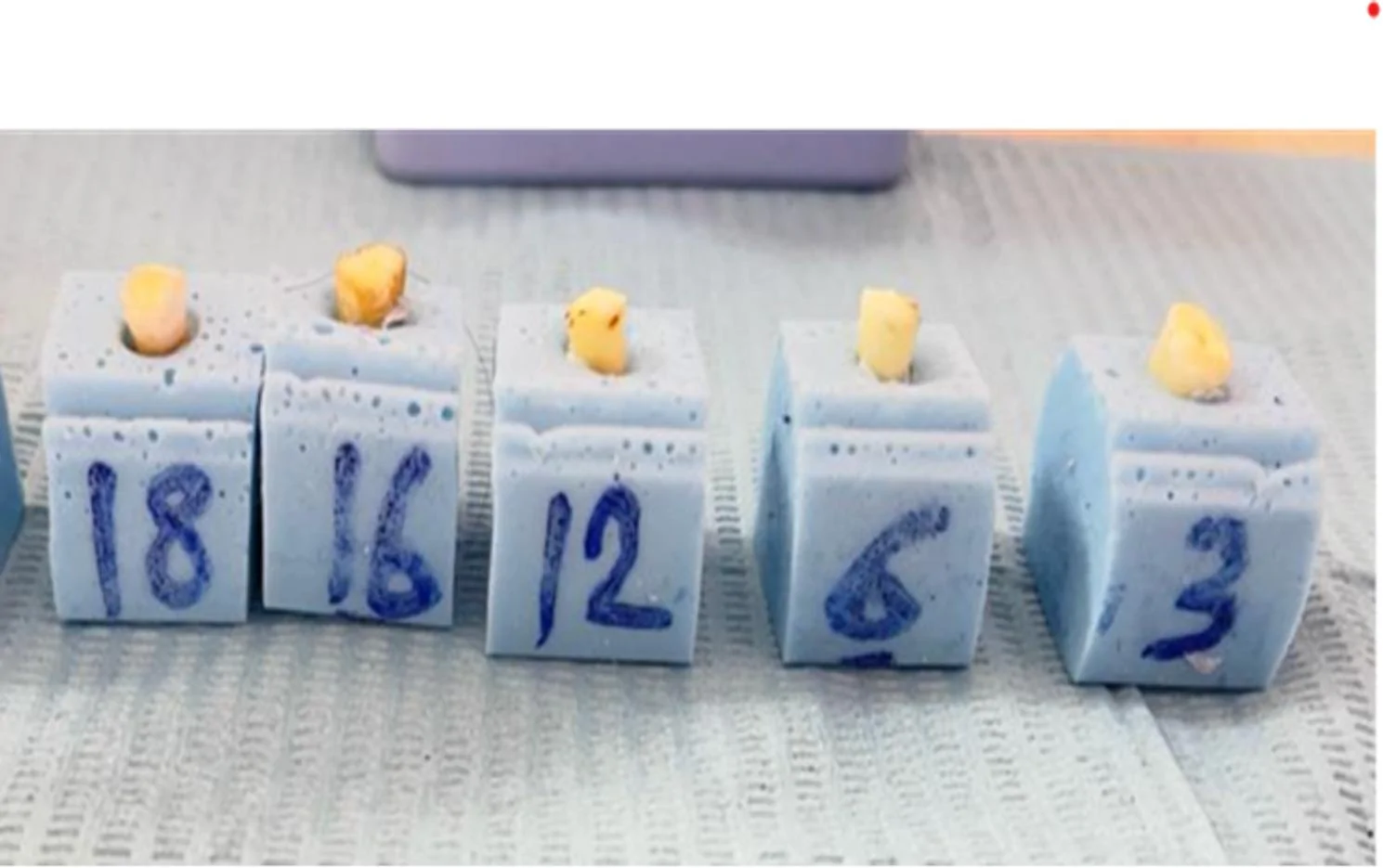
A representative photograph illustrating the teeth inserted into their corresponding blocks

Micro-brushes were used to remove debris generated from IRR cavity preparation and sequentially apply 37% orthophosphoric acid (Dia-Etch, DiaDent, Seoul, Korea) for 30 s uniformly in all samples. This additional cleaning and dentine etching step was performed to better simulate the irregular morphology of IRR cavities [24, 25]. A final wash with ten mL of saline solution was done, and absorbent paper points were used to dry the canal parts before they were put back together [26]. All preparations were performed by a single operator under standardized conditions. An elastic bandage was used to secure the teeth, and the canals were internally packed with Teflon and the teeth were externally sealed with clear epoxy adhesive (Epobond, Egypt). The specimens were remounted in silicone blocks after setting of the adhesive material for intracanal medicament placement (Fig. 2).

Teflon was removed from canals, and #40/04 paper points were used to re-dry canals. A water- based, non-setting calcium hydroxide paste (Meta Ca (OH)₂, Meta Biomed, Korea) was injected using the manufacturer’s propylene syringe tip. The needle was inserted one mm short of the WL and then gradually withdrawn during injection until it reached the canal orifice. Excess material was removed with a cotton pellet, and correct filling of the canal and resorptive cavity in each sample was confirmed radiographically in both buccolingual and mesiodistal views. Access cavities were re-sealed with Teflon tape and glass ionomer cement. Tooth specimens were incubated for seven days (37 °C and 100% humidity) in a CO₂ incubator (Heraeus, RWD, China) to simulate inter-appointment medication placement.

### Removal of medicament and grouping

After one week of Ca (OH)₂ placement, the specimens were randomized prior to the application of the experimental irrigation protocols. Randomization was performed by an investigator (resident dentist) who was not involved in specimen preparation, irrigation procedures, or outcome assessment. The 70 specimens were assigned unique identification numbers (1–70), while the seven experimental groups were coded (A–G). A computer-generated randomization sequence was generated using Microsoft Excel (Microsoft Corp., Redmond, WA, USA), and specimens were randomly allocated in equal numbers to the seven groups (*n* = 10 per group), each corresponding to a different irrigation activation technique (e.g., 41-C, 15-B). To ensure allocation concealment, group assignments were placed in sequentially numbered opaque envelopes and remained concealed until completion of specimen preparation and intracanal medicament placement.

In all groups, canals were initially rinsed using two cycles of irrigation, each involving five mL of 2.5% NaOCl for thirty seconds (total 10 mL over 60 s), followed by 5 mL of saline solution. A final flush was performed using five mL of 17% EDTA for thirty seconds. Irrigation activation was done according to each group as follows:

1. Group I (Conventional syringe irrigation, SNI) (control): Non-activated irrigation was conducted with sodium hypochlorite using a 30-gauge side-vented needle at one mm short of the WL [27]. After intermediate saline irrigation, 5 mL of 17% EDTA was flushed for 30 s without activation.
2. Group II (Manual dynamic agitation, MDA): In Group II, sodium hypochlorite was delivered following the same protocol as Group I. Manual dynamic activation was then performed using a size 40/0.04 master gutta-percha cone (Meta Biomed, Korea) inserted one mm short of the WL and agitated with 50 manual in-and-out strokes (two cycles- 30-second each) [14].
3. Group III (XP-endo Finisher file): In this group, following the introduction of sodium hypochlorite into the canal, a size 25 XP-endo Finisher file (FKG Dentaire, Switzerland) was used for irrigant agitation. The instrument was operated at 800 rpm and 1 N·cm torque, cooled prior to insertion, and then activated one mm short of the WL using gentle in-and-out strokes with a 7–8 mm amplitude (two cycles- 30-second each) [28].
4. Group IV (Sonic irrigation with EDDY tips): a tip size 25 polymer tip (VDW, Munich, Germany) mounted on a KaVo Sonic handpiece was operated at 6 kHz [8, 29]. The tip was positioned one mm short of WL and used to sonically activate NaOCl (two cycles- 30-second each).
5. Group V (Sonic irrigation with EQ-S): employed a cordless EQ-S sonic tip irrigator (Meta biomed, Korea) with a size #25 medium tip oscillating at 13,000 cycles/min ( 217 Hz) (two cycles- 30-second each) [30].
6. Group VI (Passive ultrasonic irrigation (PUI ) with IrriSafe): A stainless-steel IrriSafe ultrasonic tip irrigator (#25) (Satelec, Acteon, France) mounted on a piezoelectric unit was operated at 30 kHz and 5.5 W in an endodontic power setting [31]. The tip was placed one mm short of WL without touching canal walls and used to ultrasonically activate NaOCl (two cycles- 30-second each).
7. Group VII (Passive ultrasonic irrigation with Ultra X): An Ultra X system (Eighteeth, Changzhou, China) with a size 25 flexible X Silver tip operated at 45 kHz was inserted one mm short of WL and used to activate NaOCl (two cycles- 30-second each) [32] .

After final saline irrigation, all groups received five mL of 17% EDTA that had been activated for thirty seconds utilizing their respective irrigation techniques.

### Evaluation of residual medicament

Specimens were taken out of the silicone blocks after the activation procedures were followed, and a metallic carver was used to split the root halves. Each longitudinally sectioned half was examined under a Leica MZ12.5 stereomicroscope. Standardized digital images were acquired at 10× magnification using a digital camera attached to the stereomicroscope by a single blinded examiner.

Image analysis was performed independently using Adobe Photoshop 7.0 (Adobe Systems Inc., San Jose, CA, USA) and ImageJ software (version 1.53a, National Institutes of Health, Bethesda, MD, USA) by two additional previously calibrated and blinded examiners with more than 15 years of experience in digital image analysis.

Prior to the study, both examiners underwent calibration to standardize the image analysis criteria and measurement procedures. Inter-examiner reliability during calibration was assessed using the intraclass correlation coefficient (ICC), demonstrating excellent agreement (ICC = 0.94; 95% CI: 0.91–0.98; *p* < 0.001) according to Koo and Li [33]. Both examiners remained blind to group allocation throughout the assessment process.

The image analysis procedure consisted of two sequential steps. First, each captured image was imported into Adobe Photoshop and segmented using a semi-automatic outline selection tool. This step isolated the total root canal system from surrounding structures and subsequently divided it into the cervical, middle, and apical thirds, as well as the resorptive cavity regions.

**Fig. 3:**
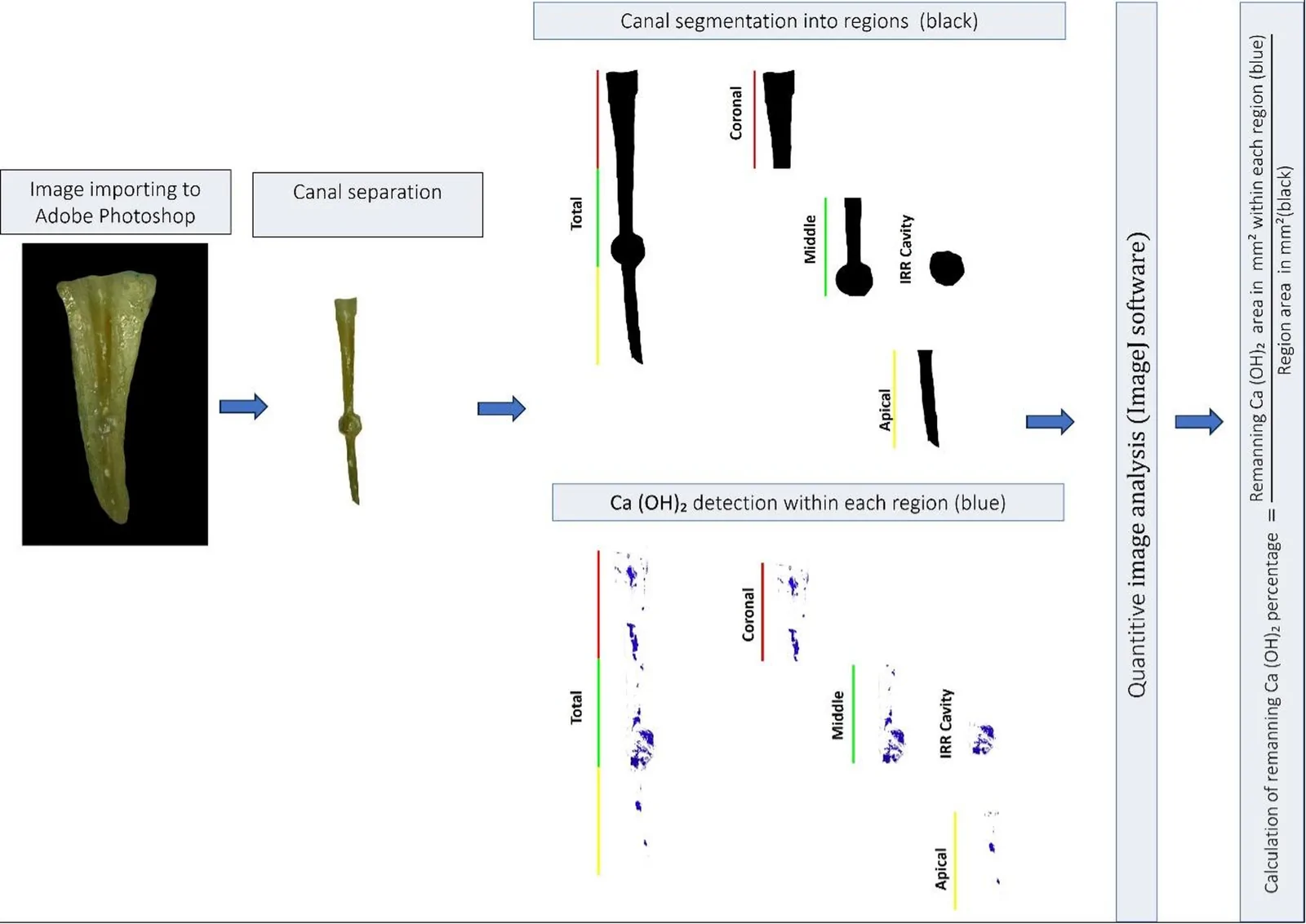
Representative image of a longitudinally sectioned tooth half illustrating the image-processing and segmentation workflow using Adobe Photoshop 7.0 and ImageJ software. Residual calcium hydroxide [Ca(OH)₂] was identified (white), segmented (blue) and quantified within the simulated resorptive cavity as well as within each root canal region (total, coronal, middle, and apical) (all delineated in black). The percentage of residual Ca(OH)₂ was subsequently calculated for the resorptive cavity and each evaluated root canal region

Areas containing residual calcium hydroxide (visualized as white), were automatically detected using Photoshop’s color range and hue/saturation adjustment tools which provide superior sensitivity and specificity in color discrimination. Detected areas were pseudo-colored in blue using the Hue/Saturation adjustment tool (Hue: 248, Saturation: 98, Lightness: -12, with “Colorize” enabled), generating a blue-highlighted image representing the spatial distribution of residual calcium hydroxide (Fig. 3).

Second, the processed images were imported to ImageJ software for quantitative analysis. A color threshold in the HSB color space was applied (Hue: 0-255, Saturation: 150–255, Brightness: 0-255 Pass bands) to the blue-colored images to automatically quantify the area of residual calcium hydroxide (mm²) within each region. The total canal area (mm²) for each region was also measured using thresholding of the canal image. The thresholding method was set to “Default,” with the threshold color set to red for visual verification against a dark background.

The percentage of residual calcium hydroxide (%) was calculated according to Taşdemir et al. [34] using the formula:

$$\%=\frac{The\:area\:of\:calcium\:hydroxide\:remanent\:in\:each\:region\:or\:in\:resorptive\:cavity\left(\:{mm}^{2}\right)}{Total\:region\:or\:resorptive\:cavity\:area\:\left({mm}^{2}\right)}\times100$$

### Statistical analysis

The statistical analysis was performed by a biostatistician who was blind to the group assignments, as all samples were labeled using the generated random codes during analysis.

Data were checked for normality using the Shapiro–Wilk test. Because the data did not satisfy the normality assumption, the final statistical analyses were performed using appropriate non-parametric methods. The overall differences among the seven activation methods were evaluated using the Kruskal-Wallis test at α = 0.05. Pairwise comparisons were performed with Dunn’s post hoc test with Bonferroni adjustment (Dunn-Bonferroni method). Intragroup comparisons among the coronal, middle, and apical levels were performed using the Friedman test followed by Dunn-Bonferroni method for pairwise comparisons. Significance was accepted at *p* ≤ 0.05 for statistical significance and *p* ≤ 0.001 for high significance. Statistical analyses and calculations were conducted utilizing GraphPad Prism version 9 (GraphPad Software, San Diego, CA, USA).

## Results

**Fig. 4:**
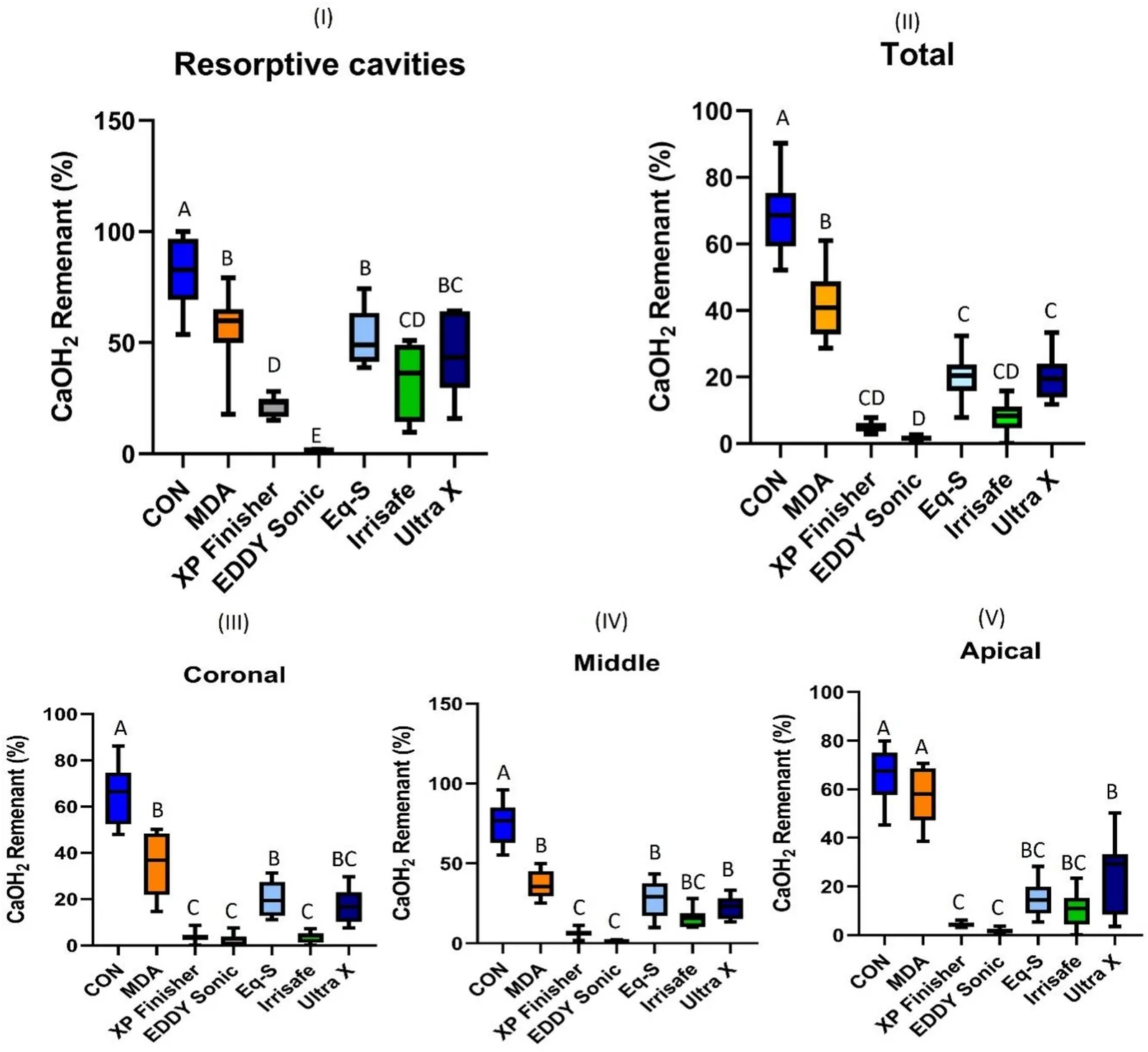
Boxplots (I–V) showing inter-group comparisons of residual Ca(OH)₂ percentages after application of the tested irrigation activation techniques in the simulated resorptive cavity (I), entire root canal system (II), and coronal (III), middle (IV), and apical (V) root canal regions. Capital letters (A–E) in each boxplot indicate the results of pairwise post hoc comparisons among irrigation activation techniques within each evaluated region. Groups assigned different letters differ significantly (*p* ≤ 0.05), whereas groups sharing at least one letter are not significantly different (*p* > 0.05)

**Fig. 5:**
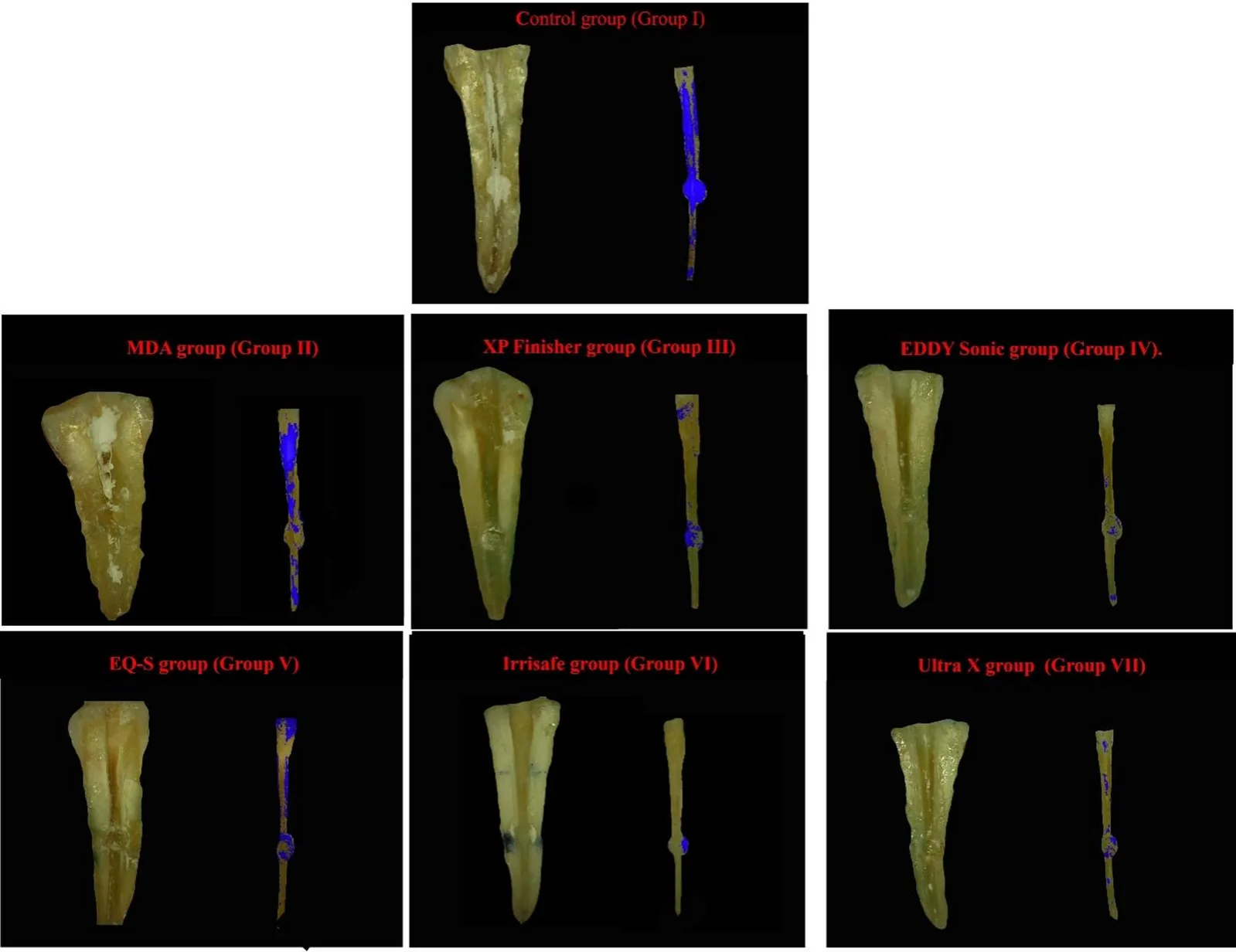
Representative stereomicroscope images illustrating the residual calcium hydroxide (blue-colored) across the experimental groups (I–VII)

The impact of various irrigation activation methods on the proportion of residual calcium hydroxide within resorptive cavities (primary outcome) and across the entire root canal regions including the coronal, middle, apical thirds (secondary outcomes) is presented in Table 1; Figs. 4 and 5.

**Table 1:** Descriptive and comparative analysis of residual Ca(OH)₂ percentages (%) across irrigation activation techniques (groups) and root canal regions. Data are presented as median (Interquartile range; Q1–Q3) and mean ± standard deviation (SD), with corresponding inter-group and intra-group comparisons

Groups	Canal region		Coronal (%)	Middle (%)	Apical (%)	*P*-value†	Total canal (%)	Resorptive Cavity (%)
	Statistic							
Control	Median (Q1–Q3)	66.48 ^Ab^ (52.4–74.63)		76.86 ^Aa^ (63.04–85.06)	67.55 ^Ab^ (57.7–75.16)	**0.042** **S**	68.66 ^A^ (59.28–75.34)	82.99 ^A^ (69.42–96.78)
	Mean ± SD	65.14±0.13.5		75.84 ± 13.82	65.64 ± 10.71		68.63 ± 11.42	81.96 ± 15.64
MDA	Median (Q1–Q3)	36.78 ^Bb^ (21.89–48.43)		35.58 ^Bb^ (29.45–45.17)	58.11 ^Aa^ (47.31–68.64)	**< 0.001 HS**	40.9 ^B^ (32.75–48.81)	59.76 ^B^ (49.97–65.16)
	Mean ± SD	34.35 ± 13.37		36.68 ± 8.54	57 ± 11.78		41.56 ± 10.46	56.45 ± 16.28
XP-Endo Finisher	Median (Q1–Q3)	3.44 ^Cb^ (2.4–4.5)		5.95 ^Ca^ (4.97–7.75)	4.2 ^Cab^ (3.57–5.18)	**0.030** **S**	4.47 ^CD^ (3.6–6.21)	23.3 ^D^ (16.61–24.83)
	Mean ± SD	3.61 ± 2.25		6.21 ± 2.58	4.43 ± 0.99		4.87 ± 1.58	21.95 ± 4.37
EDDY Sonic	Median (Q1–Q3)	3.04 ^Ca^ (0.062–3.48)		1.30 ^Ca^ (0.28–1.6)	1.47 ^Ca^ (1–2.19)	**0.199** **NS**	1.70 ^D^ (0.8–2.48)	1.48 ^E^ (0.65–1.59)
	Mean ± SD	2.72 ± 2.29		1.09 ± 0.72	1.65 ± 0.93		1.60 ± 0.95	1.25 ± 0.55
EQ-S	Median (Q1–Q3)	19.41 ^Bab^ (12.88–27.4)		29.19 ^Ba^ (17.23–37.61)	14.43 ^BCb^ (8.92–19.84)	**0.003** **S**	20.44 ^C^ (15.79–23.8)	48.96 ^B^ (41.49–63.6)
	Mean ± SD	20.21 ± 6.95		27.58 ± 11.62	15.04 ± 7.14		19.76 ± 6.59	52.92 ± 12.53
Irrisafe	Median (Q1–Q3)	4.19 ^Cb^ (1.23–5.17)		15.75 ^BCa^ (10.32–18.97)	10.99 ^BCa^ (4.5–15.26)	**0.001** **HS**	8.41 ^CD^ (4.63–11.11)	36.34 ^CD^ (14.31–49.05)
	Mean ± SD	3.48 ± 2.4		15.8 ± 5.68	10.58 ± 7.36		8.18 ± 4.74	32.58 ± 16.35
Ultra X	Median (Q1–Q3)	16.74 ^BCa^ (10.28–22.99)		23.35 ^Ba^ (15.33–28.13)	29.32 ^Ba^ (8.46–33.18)	**0.182** **NS**	19.46 ^C^ (13.91–24)	43.41^BC^ (29.64–64.18)
	Mean ± SD	17.38 ± 7.12		22.59 ± 7.10	24.86 ± 14.74		19.87 ± 6.8	43.47 ± 18.15
*P*-value‡		**< 0.001 HS**		**< 0.001 HS**	**< 0.001 HS**		**< 0.001 HS**	**< 0.001 HS**
Effect size		0.878		0.894	0.883		0.915	0.779

*‡ *Kruskal–Wallis test for intergroup comparison among irrigation activation techniques within each evaluated region (coronal, middle, apical, total and resorptive cavity). Different uppercase letters (A- E) indicate significant differences between groups within the same column (*p* ≤ 0.05) according to Dunn’s multiple comparison test with Bonferroni adjustment

† Friedman test for intragroup comparison among the coronal, middle, and apical levels within each irrigation activation group. Different lowercase letters (a-c) indicate significant differences between regions within the same group (*p* ≤ 0.05) according to Dunn’s test with Bonferroni correction

### Inter-group comparative analysis

#### Resorptive cavities

Inter-group comparisons revealed significant differences among the evaluated groups (*P* < 0.001). The control group exhibited the highest percentage (%) values relative to all experimental groups. This was followed by a cluster of significantly lower values (*P* < 0.05) comprising the MDA, EQ-S, and Ultra X groups, which showed no significant differences among themselves (*P* > 0.05). Irrisafe showed significantly lower values than MDA and EQ-S (*P* < 0.05) but remained statistically comparable to Ultra X and XP-Endo Finisher (*P* > 0.05). Conversely, EDDY exhibited the significantly lowest residual % values among all tested groups (*P* < 0.05), followed by significantly higher values in the XP-Endo Finisher and Irrisafe groups (*P* < 0.05).

#### The total canal region

The control group exhibited the highest % values, which was significantly greater than all other evaluated groups (*P* < 0.001). The MDA group exhibited a distinct, sequentially lower values (*P* < 0.05). An intermediate cluster of statistically comparable values (*P* > 0.05) was recorded by the EQ-S, Ultra X, Irrisafe, and XP-Endo Finisher groups, all of which presented significantly lower residual values than the MDA group (*P* < 0.05). Ultimately, the significantly lowest values (*P* < 0.05) were recorded in a subset comprising the EDDY, XP-Endo Finisher, and Irrisafe groups, between which no statistically significant differences were observed (*P* > 0.05).

#### Coronal, middle and apical root canal regions

Significant inter-group differences were observed across all tested root regions (*P* < 0.001), within both the coronal and middle thirds, the control group exhibited the highest values compared with all other groups (*P* < 0.05). In the coronal third, this was significantly followed by an intermediate cluster of statistically similar values (*P* > 0.05) in the MDA, EQ-S, and Ultra X groups. The significantly lowest % values (*P* < 0.05) in this region were recorded in a subset comprising the EDDY, XP-Endo Finisher, Irrisafe, and Ultra X groups, which demonstrated no significant differences among themselves (*P* > 0.05).

In the middle third, the intermediate similar cluster expanded to encompass the MDA, EQ-S, Ultra X, and Irrisafe groups (*P* > 0.05). Conversely, the significantly lowest % values (*P* < 0.05) were clustered within the EDDY, XP-Endo Finisher, and Irrisafe groups, showing statistically comparable values (*P* > 0.05).

While in the apical third, both the control and MDA groups demonstrated the highest % values, which were significantly greater than all other evaluate groups (*P* < 0.05). This was followed by significantly lower values in the Ultra X, EQ-S and, Irrisafe groups (*P* < 0.05), which showed statistically comparable values among each other (*P* > 0.05). Ultimately, the significantly lowest % values (*P* < 0.05) were recorded in EDDY, XP-Endo Finisher, Irrisafe and EQ-S groups, between which no significant differences were observed (*P* > 0.05).

### The intragroup comparative analysis (Table 1)

Within the control group, the middle third retained the statistically highest % values than the coronal and apical thirds (*P* = 0.042). Within the MDA group, the values of the apical third were significantly higher than the other two (*P* < 0.001). In the XP-Endo Finisher and Irrisafe groups, the middle and apical thirds exhibited similarly high values, while statistically significant difference was observed between the coronal and middle thirds (*P* = 0.030, *P* = 0.001 respectively). The EQ-S group showed statistically higher values at the middle and coronal thirds compared to the apical third (*p* = 0.003). In contrast, no significant difference was detected between thirds within the EDDY and Ultra X groups (*p* = 0.199, *p* = 0.182 respectively).

## Discussion

The objective of the present study was to evaluate the efficacy of various irrigation activation techniques in the removal of calcium hydroxide from the resorptive cavity and entire root canal system, given the importance of thorough medicament removal and effective cleaning of the dentinal canal walls prior to obturation, particularly in complex areas. Such cleaning is crucial for achieving an adequate seal of the obturation materials, which may contribute to the success of endodontic treatment in teeth with internal root resorption [5–7, 26].

Calcium hydroxide (Ca (OH)₂) was selected because it remains the preferred intracanal dressing for inflammatory internal root resorption (IRR) due to its hemostatic properties, ability to necrotize residual pulp tissue, and promotion of hard tissue repair, even in cases with perforating defects. These properties render it more favorable than double or triple antibiotic pastes [3, 4]. Although its aqueous formulation (Meta paste, pH 12.5) is easier to dislodge than oil-based variants [35], yet its adherence to dentine still necessitates irrigant activation [7, 18].

In the present study, the irrigation solutions and volumes were standardized for all samples, consisting of an initial irrigation with 10 mL of 2.5% NaOCl, followed by a final flush of five mL of 17% EDTA, using the same protocol across all groups, as this regimen has been reported to be effective in removing medicaments from root canals [36, 37]. Because of its potent antibacterial properties and capacity to break down organic tissue, NaOCl is commonly regarded as the gold standard for root canal irrigation [38]. Furthermore, a final rinse with EDTA, a well-known chelating agent, was performed to enhance the removal of calcium hydroxide remnants and facilitate more effective canal wall cleaning [19]. Both NaOCl and EDTA were activated according to the assigned irrigation protocol, to simulate clinical practice, in which both irrigants are commonly used sequentially during final irrigation [29, 39–41]. Nevertheless, the independent contribution of each irrigant to calcium hydroxide removal cannot be distinguished from the present study design. Therefore, the current findings should be interpreted as reflecting the overall efficacy of the complete irrigation protocol rather than the isolated effect of activation during NaOCl or EDTA irrigation alone.

Although artificial plastic root canal models with standardized grooves or simulated internal resorption cavities have been used previously to evaluate Ca(OH)₂ removal, these models do not accurately replicate the nature and morphology of the human root canal system [28, 42, 43]. Therefore, the present in vitro study utilized 70 extracted human single-rooted premolars in which standardized cavities were mechanically prepared in accordance with previously reported methodologies [23, 24, 26, 29, 40]. This model is widely employed due to its high level of standardization, reproducibility, and inter-sample comparability, thereby enabling reliable evaluation of irrigation protocol efficacy under controlled laboratory conditions [1, 37, 44]. The standardized positioning of the simulated IRR cavities was carefully selected, and the cavities were etched with 37% orthophosphoric acid to replicate surface irregularities [24, 25]. This approach enhances the resemblance of artificial model to IRR lesions and promotes calcium hydroxide retention, thereby simulating the difficulty of medicament removal [24, 25, 29]. As the phosphoric acid conditioning protocol was applied uniformly across all groups, it could not have biased the between-group comparisons. Crown preservation provides a reservoir for irrigants, which may enhance irrigant dynamics and penetration into inaccessible areas during activation, thereby allowing a more clinically relevant assessment of medicament removal [45, 46].

Various techniques have been widely employed in endodontic studies to evaluate residual intracanal medicaments, including digital photography [47, 48], operating microscopy (OM) [29], stereomicroscopy [18, 49], scanning electron microscopy (SEM) [50], and micro-computed tomography (micro-CT) [51]. In the present study, stereomicroscopy combined with digital quantitative image analysis was used because it provides a measurable, reproducible, and cost-effective method for assessing residual calcium hydroxide while reducing the subjectivity associated with scoring-based evaluations [37, 52].

Adobe Photoshop was used for the initial image segmentation prior to ImageJ analysis because direct thresholding of the original images was challenging due to the similar color and brightness characteristics of residual calcium hydroxide and surrounding dentin. Photoshop’s color-based selection and hue/saturation adjustment tools facilitated more accurate isolation of the residual medicament before quantitative measurement [53]. The processed images were subsequently analyzed in ImageJ using a standardized thresholding protocol to ensure objective and reproducible area quantification [26].

To minimize measurement bias and strengthen the internal validity of the study outcomes, sample size calculation and strict standardization procedures were implemented, including standardized specimen preparation, sectioning, image acquisition, and blind image analysis. Nevertheless, this method provides only a two-dimensional assessment and does not permit a three-dimensional volumetric evaluation of residual material within the resorptive cavity [32]. Although micro-CT offers a more comprehensive three-dimensional analysis, its application is limited by high cost and restricted availability [18, 54].

Generally, the key finding was a clear hierarchy among activation methods: polymer-based sonic activation (EDDY), adaptive XP-Endo NiTi rotary Finisher and Irrisafe passive ultrasonic activation (PUI) showed superior effectiveness throughout the entire root canal system. Specifically, EDDY demonstrated the lowest residual medicament within resorptive cavities. Consequently, the null hypothesis asserting equal performance among irrigation activation techniques in different canal regions was rejected.

Hydrodynamically, it was suggested that the activation tip flexibility, design and oscillation amplitude critically influence fluid velocities and shear stress within root canal systems [7, 8, 11]. In the present study, the enhanced cleaning efficacy of EDDY sonic activation, particularly within resorptive cavities, may be explained by mechanisms previously reported in the literature [11, 12]. These include high-amplitude oscillation, effective acoustic streaming, and disruption of the vapor lock effect, in addition to the capacity of its flexible polymeric tips to better adapt to inaccessible areas and to sustain a three-dimensional oscillation upon contact with canal walls. Collectively, these features may potentially enhance irrigant penetration and promote more uniform removal of medicament in challenging canal anatomies [29, 55–57]. In contrast to the flexible EDDY sonic tip, which has no effect on the streaming velocity during contact, the probability of the ultrasonic stainless-steel rigid tip coming into contact with the canal wall dampens the process of fluid movement along the root canal wall (despite its higher frequency) [13, 29]. Although EQ-S employs bendable polymer tips, it demonstrated lower efficiency than EDDY sonic and passive ultrasonic irrigation in the current study, likely due to its lower operating frequency (217 Hz) [30, 58, 59].

These findings, however, run counter to earlier studies that employed scoring-based assessment methods and reported comparable efficacy of EDDY and passive ultrasonic irrigation in removing Ca(OH)₂ from artificially created IRR cavities [23, 26, 60]. Marques-da-Silva et al. [29] used different imaging modalities (endodontic OM and SEM) and a higher concentration of NaOCl (5%) and reported no significant difference between EDDY and XP-Endo Finisher in the removal of Ca(OH)₂ from simulated IRR defects.

The present study’s findings showed also that the XP-Endo Finisher and Irrisafe ultrasonic irrigation were the second most effective techniques for enhancing calcium hydroxide removal from simulated IRR cavities, exhibiting comparable efficacy. This comparable efficacy is consistent with the findings of Sarıyılmaz et al. [23], Wigler et al. [28], Keskin et al. [39], and Arora et al. [48].

It has been reported that the XP-Endo Finisher possesses the unique ability to transform into its austenitic phase having a curved (S) three-dimensional shape at intracanal temperature, enabling the file to adapt three-dimensionally and achieve enhanced contact with canal walls, particularly in irregular anatomical areas that are inaccessible to conventional instruments like IRR cavities [21, 40]. While the cleaning efficacy of Irrisafe ultrasonic irrigation may be attributed to increased irrigant velocity and enhanced hydrodynamic effects. Acoustic streaming and cavitation may further promote continuous irrigant exchange, thereby improving irrigant penetration and calcium hydroxide removal in canal irregularities such as resorptive defects [12, 41, 60].

Nevertheless, the present results are inconsistent with those of a previous study reporting superior performance of the XP-Endo Finisher compared with Irrisafe passive ultrasonic irrigation and sonic activation using the EndoActivator system [40]. That study utilized 3% NaOCl and 2 mL of EDTA for medicament removal from smaller simulated IRR cavities (1.4 mm in diameter and 0.7 mm in depth) than those used in the present investigation. While the lower efficacy of ultrasonic activation compared with XP-Endo Finisher reported by Marques-da-Silva et al. [29] may be attributed to the use of a rigid ultrasonic tip with a 30/0.02 taper. Campos et al. [21] reported superior outcomes with XP-Endo Finisher in IRR cavities compared with a different ultrasonic system (Irrisonictip, Helse, Brazil). The study employed an alternative IRR cavity preparation protocol using 5% nitric acid and 8% sodium hypochlorite, as well as a different irrigation regimen involving NaOCl followed by EDTA and a final NaOCl rinse. In contrast, Donnermeyer et al. [60] reported superior performance of ultrasonic irrigation in artificial grooves compared with the XP-Endo Finisher. This discrepancy may be related to the use of a smaller irrigant volume (3 mL) and a lower concentration of sodium hypochlorite (3%), as well as the absence of EDTA chelation during irrigation Taken together, the observed discrepancies are likely attributable to methodological variations, including differences in scoring criteria and calcium hydroxide formulations used across studies.

Interestingly, although Ultra X ultrasonic activation yielded significantly lower Ca(OH)₂ removal from IRR cavities compared with the XP-Endo Finisher in the present study, this difference was not statistically significant when compared with Irrisafe in IRR cavities or with both XP-Endo Finisher and Irrisafe in terms of overall canal performance. Nevertheless, this is in contrast to the improved performance of Ultra X tips reported by Güven et al., [59] and Adl et al. [32], in removing medicaments from artificially created grooves than other sonic and ultrasonic activation techniques (EndoActivator, Endosonic Blue, and size 25 K ultrasonic file connected on a piezoelectric handpiece). The inconsistency across studies underscores the influence of tip design nuances, ultrasonic frequency, and IRR model on cleaning efficacy, highlighting the need for standardization in methodological approaches across future research.

When considering calcium hydroxide removal from the entire canal system in the present study, conventional syringe irrigation showed pronounced shortcomings, leaving large volumes of calcium-hydroxide residue. This finding corroborates experimental evidence that the vapor-lock phenomenon traps air within root canal lumen and impedes irrigant exchange when only passive needle delivery is used [7]. Similarly, MDA provided only modest improvement over syringe in the coronal and middle thirds as well as within the internal resorptive (IRR) areas, while showing comparable efficacy to syringe irrigation in the apical third, where high Ca(OH)₂ remnants were observed. This finding is consistent with earlier studies by Khare et al. [61] who attributed this limited efficacy to the low-frequency mechanical agitation generated by manual techniques. Additionally, the tapered anatomy of the root canal may facilitate apical compaction of Ca(OH)₂ during its removal, while the restricted irrigant volume and fluid flow within the narrow apical region may further compromise medicament removal compared with the wider coronal portion of the canal [14, 17, 35, 36]. On the other hand, EQ-S, Ultra X, Irrisafe, and XP-Endo Finisher demonstrated comparable performance, with all four systems achieving superior Ca(OH)₂ removal from the entire root canal compared to MDA. This finding indicates that activation-based irrigation techniques are more effective than manual agitation in enhancing overall medicament removal throughout the canal space [35, 36]. Notably, EDDY, XP-Endo Finisher, and passive ultrasonic irrigation exhibited the highest and comparable efficacy across the entire root canal system, suggesting that these techniques may be particularly effective when comprehensive three-dimensional irrigant agitation is required, especially in root canals with internal resorptive (IRR) defects.

The relatively uniform removal of Ca(OH)₂ across the canal thirds observed with the EDDY and Ultra X systems in the present study may be explained by their ability to enhance fluid dynamics. By improving irrigant exchange, agitation, and acoustic streaming, these activation techniques may facilitate more uniform irrigant distribution throughout the root canal system [7].

Nevertheless, the current study acknowledges certain limitations that warrant careful consideration. The prospective sample size was based on the primary omnibus comparison, whereas Bonferroni-adjusted pairwise comparisons were secondary post hoc analyses and were not specifically powered a priori. Nevertheless, significant differences were detected in the key pairwise comparisons, indicating that the selected sample size was adequate despite the conservative Bonferroni adjustment. In addition to the limitations associated with the two-dimensional image analysis employed, previous studies have demonstrated that curved roots harbor three-to-four-fold larger Ca(OH)₂ residues than straight canals, accordingly the straight, single-rooted tooth model may underrepresent clinical difficulty [54]. Oil-based Ca(OH)₂ formulations have been reported to adhere more tenaciously and are significantly harder to dislodge than water-based Ca(OH)₂ tested in the present study [62]. Moreover, irrigant volume, concentration and temperature were fixed, even though a previous work indicates that surfactant addition or pre-heating NaOCl markedly alters penetration depth and tissue-dissolution capacity [38]. Finally, the use of a single irrigation dynamic protocol and the lack of direct fluid dynamics measurement represent limitations of the study, as both factors may influence calcium hydroxide removal efficacy [7].

Future investigations should therefore employ three-dimensional volumetric micro-CT analysis to capture hidden residues located within lateral extensions in curved or multirooted systems to validate activation efficacy under realistic anatomy; compare water- and oil-based Ca(OH)₂ as well as antibiotic-containing pastes to broaden generalizability; and optimize irrigant chemistry (e.g., heated or surfactant-enhanced NaOCl), as well as irrigant volume, concentration, and activation protocols. Critically, randomized clinical trials assessing patient-centered outcomes, post-operative pain and periradicular healing are needed.

## Conclusion

Complete removal of Ca (OH)₂ paste from internal resorption cavities remains elusive; nevertheless, the present study shows that activation protocols based on high-amplitude polymeric sonics (EDDY) effectively reduce residual medicament from resorptive cavities when compared with conventional syringe irrigation or the other activation devices evaluated. On the other hand, EDDY, XP-Endo Finisher, and passive ultrasonic irrigation (PUI) demonstrated comparable efficacy and were the most effective techniques for calcium hydroxide removal throughout the entire root canal system.

## Data Availability

All the data during the current study (including the ImageJ measurement outputs and processed image-derived data) are available from the corresponding author on reasonable request.
